# Targeted therapy using nanocomposite delivery systems in cancer treatment: highlighting miR34a regulation for clinical applications

**DOI:** 10.1186/s12935-023-02929-3

**Published:** 2023-05-06

**Authors:** Muhammad Javed Iqbal, Zeeshan Javed, Haleema Sadia, Sajid Mehmood, Ali Akbar, Benish Zahid, Tariq Nadeem, Sadia Roshan, Elena Maria Varoni, Marcello Iriti, Eda Sönmez Gürer, Javad Sharifi-Rad, Daniela Calina

**Affiliations:** 1 Department of Biotechnology, University of Sialkot, Punjab, Pakistan; 2 Centre for Applied Molecular Biology, University of the Punjab, Lahore, Pakistan; 3 BUITEMS, Quetta, Pakistan; 4 Department of Biochemistry, Islam Medical and Dental College, Sialkot, Pakistan; 5 Department of Microbiology, University of Balochistan Quetta, Quetta, Pakistan; 6 Department of Pathobiology, KBCMA, CVAS, Sub Campus University of Veterinary and Animal Sciences, Narowal, Pakistan; 7 National Centre of Excellence in Molecular Biology, University of the Punjab, Lahore, Pakistan; 8 Department of Zoology, University of Gujrat, Gujrat, Pakistan; 9 Dipartimento di Scienze Biomediche, Chirurgiche ed Odontoiatriche, Università degli Studi di Milano, Milan, Italy; 10 Department of Agricultural and Environmental Sciences, Università degli Studi di Milano, Milan, Italy; 11 Faculty of Pharmacy, Department of Pharmacognosy, Sivas Cumhuriyet University, Sivas, Turkey; 12 Facultad de Medicina, Universidad del Azuay, Cuenca, Ecuador; 13 Department of Clinical Pharmacy, University of Medicine and Pharmacy of Craiova, Craiova, 200349 Romania

**Keywords:** miR34a, tumorigenesis, nano-delivery systems, cancer treatment, p53 signaling

## Abstract

The clinical application of microRNAs in modern therapeutics holds great promise to uncover molecular limitations and conquer the unbeatable castle of cancer metastasis. miRNAs play a decisive role that regulating gene expression at the post-transcription level while controlling both the stability and translation capacity of mRNAs. Specifically, miR34a is a master regulator of the tumor suppressor gene, cancer progression, stemness, and drug resistance at the cell level in p53-dependent and independent signaling. With changing, trends in nanotechnology, in particular with the revolution in the field of nanomedicine, nano drug delivery systems have emerged as a prominent strategy in clinical practices coupled with miR34a delivery. Recently, it has been observed that forced miR34a expression in human cancer cell lines and model organisms limits cell proliferation and metastasis by targeting several signaling cascades, with various studies endorsing that miR34a deregulation in cancer cells modulates apoptosis and thus requires targeted nano-delivery systems for cancer treatment. In this sense, the present review aims to provide an overview of the clinical applications of miR34a regulation in targeted therapy of cancer.

## Introduction

MicroRNAs (miRNA) are highly conserved, non-coding, complex, and evolutionary short RNA molecules that are responsible for multiple functions. These are 18–25 nucleotide length molecules that regulate gene expression by inhibiting genetic translation and significantly decreasing mRNA stability, usually through premature base pairing complementary to the 3΄-untranslated region [1]. miRNAs possess anticancer activity and are highly accepted practicable therapeutic candidates to knock down oncogene expression in humans and mouse models [2]. It has been observed that miRNAs biologically act as tumor suppressors or oncogenic RNAs regulators and are believed to have a key role in targeted therapy in the future [3]. OncomiRs are oncogenic miRNAs aberrantly upregulated in cancer cells and significantly contribute to the onset of carcinogenesis by inhibiting tumor suppressor genes that normally prevent cancer development by blocking proto-oncogenic expression [4]. miRNAs have proven to be one of the valuable expression treatment strategies for silencing OncomiRs with miRNA inhibitors. For example, miR-155 has been reported as a significant OncomiR, and its oncogenic role has been observed in a variety of hematologic and solid tumors. The miR-155 overexpression in lymphoid tissues induces aggressive lymphoma in a miR-155 Cre-loxP tetracycline-regulated knock-in mouse model [1, 3]. Several studies have addressed the epigenetic downregulation of miR34a among various cell lines, including lung, breast, liver, colon, bladder, kidney, cervical, prostate, and oral cancer, as also in primary melanoma and leukemia [5]. However, CpG methylation of miR-34b/c has also been reported in colorectal cancer, squamous cell carcinoma, and malignant melanoma, while deregulation of miR34a has been stated in various experiments. Taken together, the miR-34a/b/c inactivation and its related downstream targeted molecular signaling cascades are the significant contributing factors to the onset of tumorigenesis [6, 7]. Indeed, the miRNA therapeutic strategy is currently considered the most attractive option for the scientific community to grab abnormal cancerous cell dynamics, being widely assessed in preclinical trials as miRNA replacement therapy. In this line, miR34a was the first reported as having tumor suppression potential [8], with low miR34a expression level being reported to be linked to large tumor size [9]. The advances stated in miR34a biology have successfully attained the hype of interest among biopharmaceutical companies to design therapeutically-effective candidates to conquer the temple of molecular oncology. For instance, in lung carcinoma, miR34a has been investigated as a replacement therapy candidate. The exogenous delivery of miR34a in clinical trials showed a massive reduction in tumor growth, with the loss of miR34a being considered as the key contributing factor towards the aggressive behavior of lung cancer stem cells. Thus, this emerging replacement therapy involves the exogenous delivery of miR34a to mimic and restore miR34a activity in cancer models [10, 11]. Nonetheless, despite the recent biological advances to tackle cancer pathogenesis, it remains untreatable and demands the need for novel therapeutic tools to target culprit cancer-causing cells at the nanoscale level. During the last decade, nanobiotechnology has emerged as one of the most fascinating tools to tackle multifaceted and complex cancer cells [12]. It attains a rapid gain of interest in the scientific community by designing nano-sized delivery systems that act as anticancer agents and are biopharmaceuticals injected into the cells by systematic administration [13]. Thus, to facilitate the shuffling of these emerging delivery systems from the bench to the bedside, they should be stable, safe, specific, and effective. In this sense, the present review article aims to cover the most important aspects of the clinical application of miR34a in targeted cancer therapy, with special emphasis on its use through nanocomposite delivery systems.

## Review methodology

This updated review analyzed the tumor suppressor function of miR-34 in a p53-independent and p53-dependent manner. The use of nanoparticles to deliver potential anticancer therapies with miR-34 RNA was also analyzed. To achieve these objectives, specialized databases such as PubMed/MedLine, Scopus, TRIP database, Baidu Scholar, and ScienceDirect were searched using the following MeSH terms: “Apoptosis/genetics”, “Cell Cycle Checkpoints/genetics”, “Cell Proliferation”, “Cell Transformation, Neoplastic/genetics”, “Drug Carriers/chemistry”, “Nanomedicine/trends”, “Nanoparticles/administration&dosage”, “Nanocomposites”, “Nanotechnology/trends, “Drug Delivery Systems”, “Gene Expression Regulation”, “Neoplastic/genetics”, “Neoplasms/drug therapy”, “MicroRNAs/genetics”, “MicroRNAs/metabolism”, “Polymers/chemistry”, “RNA-Binding Proteins/genetics”, “Tumor suppressor protein p53/deficiency”, “Tumor Suppressor Protein p53/genetics”, “Tumor suppressor protein p53/metabolism”. The most important data have been summarized in figures and a table.

## miR34a biogenesis and gene regulation

**Fig. 1:**
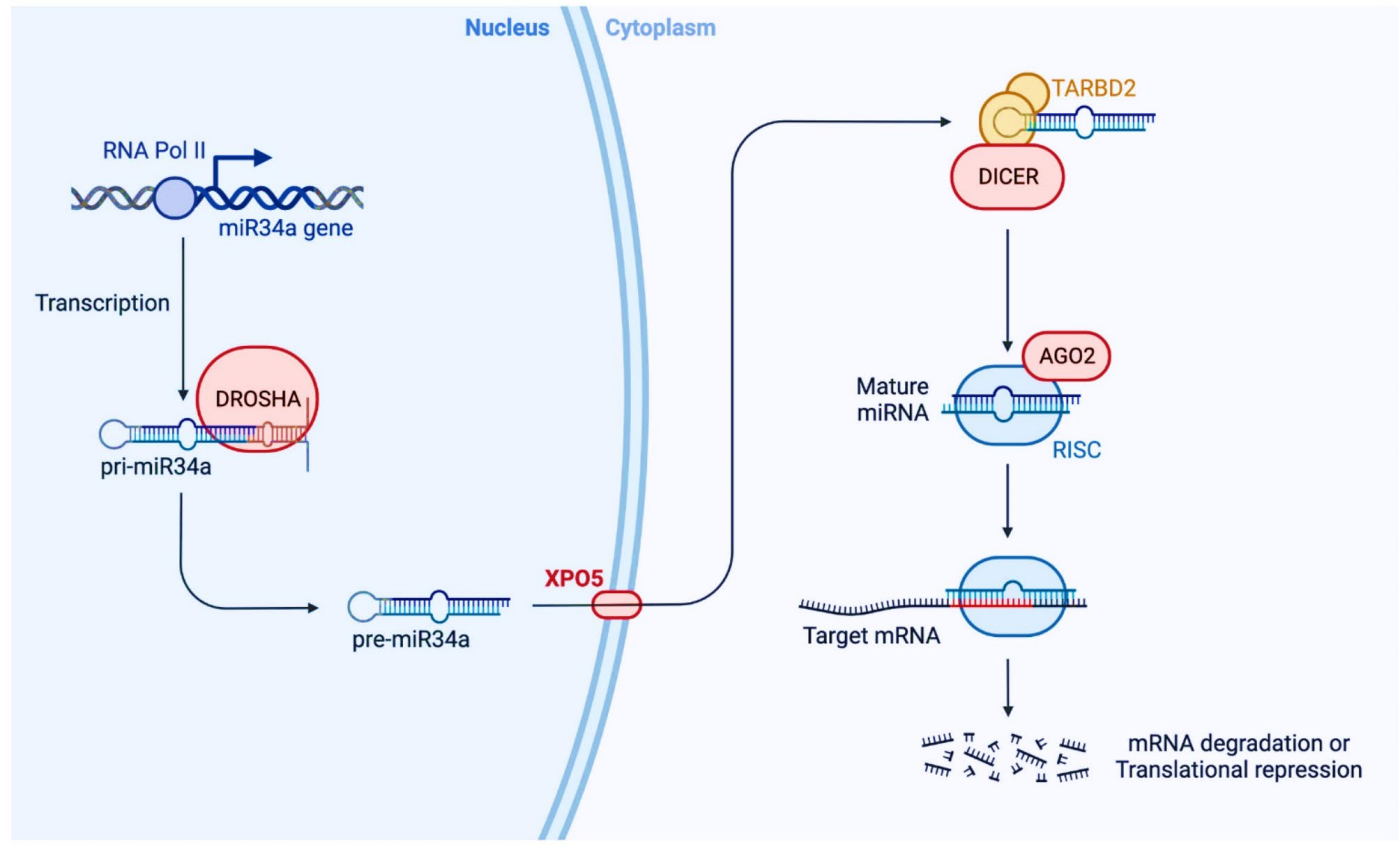
Diagram with molecular mechanism of miR34a biogenesis and its mode of action to target specific mRNA molecules to regulate gene expression in the human biological system. miR34a are usually transcribed by the enzyme RNA polymerase II, reported to bind with the DNA promoter region to form a long hairpin molecule, primary miRNA (pri-miRNA). Further, within the nucleus, micro-processing is done and pri-miRNA is cleaved by human RNase III Drosha to about 70 nt long stem-loop molecule known as precursor miRNA (pre-miRNA). Pre-miR34a is exported from the nucleus to the cytoplasm by a shuttle protein exportin-5 (XPO5). In the cytoplasm, it is further processed by another human RNA III Dicer and TAR RNA-binding protein 2 (TARBP2) to generate mature miR34a to about 22 nt long mature strand, which is loaded into the RNA-induced silencing complex (RISC). After incorporation of the miR-34a-RISC complex, miR34a guides the complex to bind with the 3΄ UTR region of targeted mRNA partially or entirely and thus play a decisive role in the regulation of target gene expressions

miR34a has been functionally described as the master regulator of tumor suppressors, with large evidence suggesting that miR34a is involved in the regulation of gene expression at the post-transcription level, thus directly or indirectly shifting both the stability and translation capacity of mRNAs [14]. The human genome encodes < 1000 miRNAs playing a decisive role in the regulation of most genes controlling translation. Briefly, the miR34 family has 3 important members, miR34a, miR34b, and miR34c. In humans and mice, miR-34a has been ubiquitously expressed in normal tissues, however, miR-34b and miR-34c are profoundly expressed in the brain, testes, fallopian tubes, lung tissues, and other specific tissues. In humans, the miR-34a gene is located at chromosome 1p36.2, while miR-34b and miR-34c are expressed from a common transcript of chromosome 11q23 [15] (Fig. 1).

## Regulation of miR34a via p53 signaling

Transcriptional expression of miR34a is regulated by p53, whose activation is observed to halt the cell cycle proliferation by inducing cell damage repair or apoptosis. Researchers have found that the 5΄ upstream region of miR34a is the selective binding site for p53 upon DNA damage or cell stress [16]. Upon genotoxic stress, miR34a upregulation is strongly induced in a p53-dependent manner. In various studies on cancer models of lung, pancreas, colon, and breast cancer, it has been established that mutation in p53 negatively regulates miR34a expression, however, p53-independent regulation of miR34a is also reported in several molecular studies. Currently, molecular evidence supports that miR34a overexpression inhibits cell proliferation and induces cell senescence, and its expression level is responsible to decide the cell fate [17, 18]. Moreover, both MDM2 and MDMX/4 are p53-binding proteins, and recent studies have highlighted the biological interplay of MDM2 and MDMX/4 proto-oncogenes with the p53 tumor suppressor protein. It has been noted that MDM2 and MDMX/4 induce p53 degradation by interacting with the C-terminal regulatory domain of p53, ultimately suppressing apoptosis [19]. Also, molecular investigation in miR34a knockout (KO) mice models showed that it prominently affects infertility, and triggers post-natal mortality, fibrotic diseases, breast abnormality, invasive carcinoma, and multiple lung-related complexities [20, 21].

**Fig. 2:**
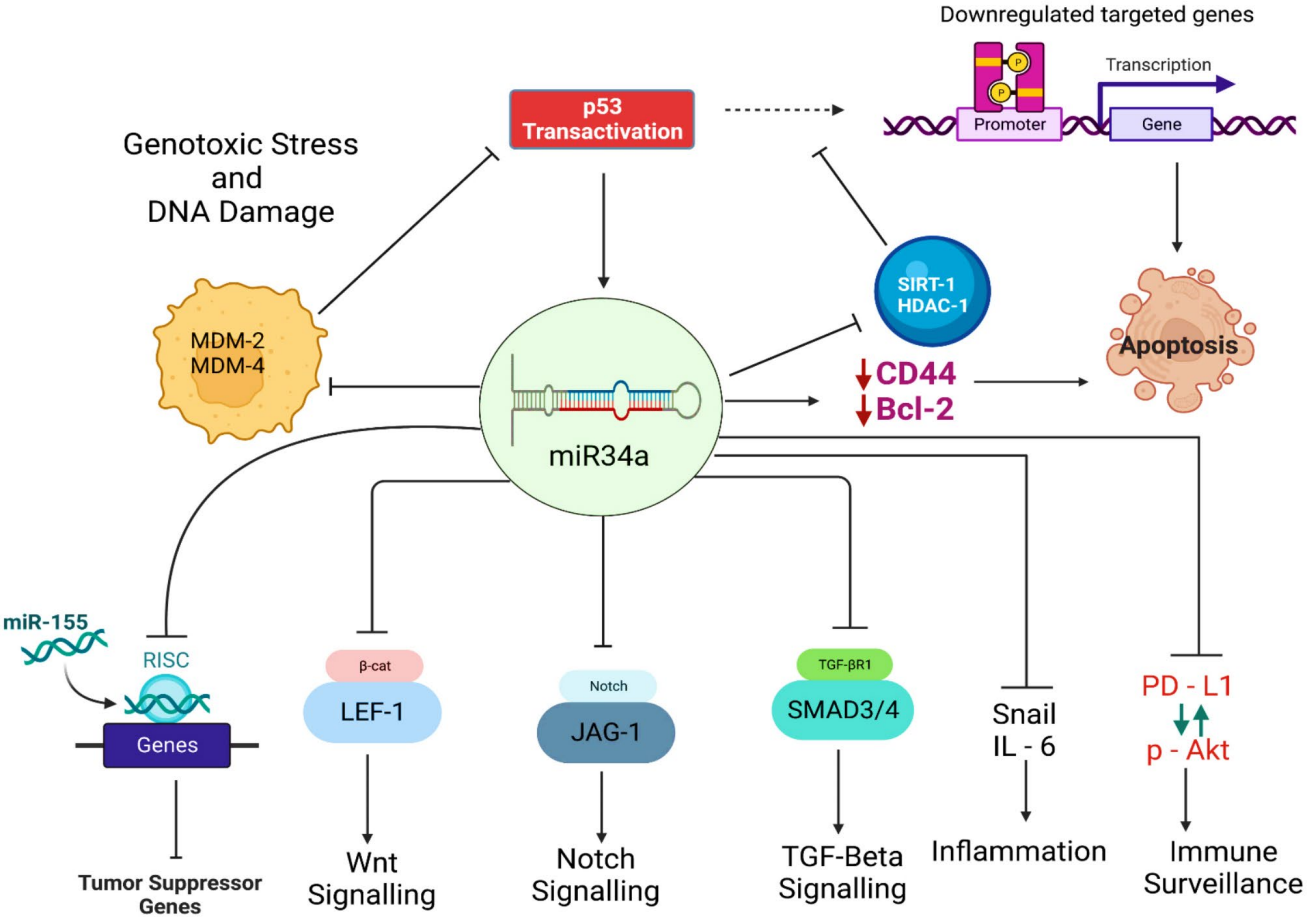
Diagram regarding p53-dependent regulation of miR34a: upon DNA damage and cellular stress, p53 binds at the specific promoter region of miR34a and promotes the transcriptional activation of miR34a. In carcinogenesis, miR34a protect p53 via the various feedback loop and upregulation of miR34a inhibits cellular proliferation and induces senescence by targeting various signaling cascades. Abbreviations and symbols: ↑increase, ↓decrease, Silent information regulator 1 (Sirt1), Histone deacetylase 1 (HDAC1), Deoxyribonucleic Acid (DNA), Lymphoid enhancer factor 1 (LEF1), Notch extracellular subunit (Notch), Jagged-1 (JAG1), Transforming growth factor-β (TGF-β), Interleukin (IL), programmed cell death ligand 1 (PD-L1).

On the other side, both histone acetylation and de-acetylation have a profound influence on the regulation of eukaryotic gene expression. For instance, SIRT-1, a histone deacetylase is clinically observed to play a contributing role in the pathogenesis of many cancers. Recently, SIRT-1 has been stated as an oncoprotein in a variety of cancers, including lymphoma, breast, lung, ovarian, pancreatic, and bladder cancer. Briefly, SIRT-1 directly deacetylates the p53 protein and deregulates the biological mechanism of controlling cell division, “apoptosis” [22]. Similarly, the HDAC-1 gene encodes deacetylase protein as a core component of the histone deacetylase complex, which is reported to target tumor suppressor proteins, including p53, and Rb genes, and modulate their biological activity of cell growth and apoptosis [23]. miR34a inhibits the SIRT-1 and HDAC-1 histone deacetylase activity and is thus reported to induce p53 transactivation and its downstream signaling cascade to limit an abnormal division of cancer cells [24]. CD44 is a cell surface molecule that is involved in signaling for cell surveillance and also exerts a prominent role in regulating complex biological processes, including cell proliferation, differentiation, migration, angiogenesis, and cytokines production. CD44 is, thus, a vital biological entity associated with the pathogenesis of cancer, and miR34a has been found to have a robust association with cancer stem cell regulation in various cancer types [25]. Specifically, miRNA deregulation has been implicated in tumor progression and metastasis, with miR34a, a p53 target, under-expressed in CD44 + prostate cancer cells [26]. Indeed, miR34a decisively represses CD44 to inhibit prostate cancer stem cells and metastasis, indicating the direct involvement and pivotal role of both CD44 and miR34a in cancer progression versus cancer prevention [27]. Also, researchers validated CD44 as a potentially functional target of miR34a, where the CD44 knockdown by miR34a over-expression leads to the inhibition of cancer cell regeneration and metastasis [25]. Finally, miRNA also plays a pivotal role in coupling and antagonist with Dicer-1, whose downregulation leads to metastasis induction. Experiments in animals have shown that in colon cancer cells, the Dicer-1 deficiency is reported to be responsible for the over-expression of CD44, while Dicer-1 deregulation in human and other mice models is believed to be linked with defective production of miR34a [28] (Fig. 2).

## Interplay of miR34 with critical biological targets

The guardian of the human genome “Onco-suppressor p53” also relies on multiple supporting biomolecules to fulfill their cellular surveillance mechanisms [29]. Recently, in both xeno-grafted mouse models and in vitro analysis, researchers stated miR34a for therapeutic purposes, quite hopeful in downregulating both NOTCH-1 and Bcl-2 expression and potentially increasing the apoptotic mechanisms. The NOTCH deregulation is associated with an increase in apoptosis, similar to Bcl2 [30]. Also, miR-34a is a key regulating player in programmed cell death initiation triggered by the activation of p53 genes. For example,  Tsungeng Chang et al. [31] performed a series of experiments using cells with wild and mutant type p53 genes separately, to critically analyze the expression of miR-34a at the cellular level. As the main findings, the authors stated that cells with wild-type p53 have a high expression than the mutated form of p53 [31]. The induction of synthetic miR-34a in both wild and mutated cells revealed that a higher apoptosis rate was observed in the wild type having functional p53 gene compared to mutated cells. These results revealed the dependency of miR-34a-regulated apoptosis on p53 gene functionality. Also, such experiments have shown that the leading cause of cancer in pancreatic cells was the reduced expression of miR-34a so the key function of miR-34a is the regulation of gene expressions proposed by the p53 gene [31].

In cancer biology, the tumor suppressor protein p53 is the central modulator to initiate a p53-dependent transcriptional feedback network upon genotoxic stress by modulating miRNA expression [31]. Clinical trials of miR-34a replacement therapy for cancer have been done in various experimental studies and it has been noted that p53 is responsible for controlling the miR34a expression, which is deregulated in carcinogenesis. Under such biological conditions, the exogenous expression of miR34a with defected p53 tumor suppressor pathway is a crucial obstacle for miR34a-based therapeutics. Therefore, the alternative p53-independent strategies to stabilize miR-34a expression attained huge clinical acceptance and efforts have been done to observe the miR-34a upregulation by various p53-independent biological processes [32]. Such dynamic p53-independent expression of miR34a can be supervised in couples with a p53-dependent mechanism or deregulated p53 cells. These independent biological regulators could be intrinsic and extrinsic signaling cascades [33]. Tumor suppressor miRNA, miR34a, is believed to be frequently depleted in cancer tissues and it is associated with the genetic expression of multiple signaling pathways, including NOTCH-1, CD44, Bcl-2, Myc, Met, CDK-4/6, and various other biological molecules [33]. Moreover, miR34a transcription is clinically proved to be pre-dominantly triggered by p53 via binding affinity of p53 binding domains/sites on the promoter region of miR34a upon DNA damage [34, 35]. Another biological way to trigger miR34a expression is through multiple feedback loops. Indeed, ample molecular evidence in clinical studies has determined the induced expression of miR34a in p53-deficient cells and in mice models that support the alternative p53-independent strategies of miR34a expression [36]. Thus, due to the engagement of multiple feedback loops, it is necessary to consider the disrupted effect of p53 for the optimal formulation of a miRNA-based anti-cancer drug designing and delivery system. From data available to date, miR34a is reported to target around 700 genes to regulate cell proliferation (NOTCH-1, MDMX, Myc-N), apoptosis (Bcl-2, SIRT-1, BIRC-5), senescence (E2F3), cancer stemness (CD-44, Nanog, Sox-2), immunity (PD-L1, DGK), and where the downregulation of miR34a have evidenced a strong molecular association with cancer onset [33]. However, in few cases, deletion of the genome locus of chromosome 1p36 responsible for encoding miR34a transcript has also been reported in different cancers [40].

## miRNA therapeutics for cancer

MicroRNA-based therapies for cancer treatment are believed to restore the body’s innate potential to fight against cancer onset by downregulating the expression of targeted oncogenes. Among these, MRX34 is one of the leading therapeutic product candidates of MiRNA that has made some advancements in the clinical testing of cancer patients as the first microRNA replacement therapy. The miRNA-34 family was first identified in 2007 as a direct target of p53. In 2011, the first anti-miRNA enters into a clinical trial as an anti-hepatitis drug. In 2013, this highly appreciated entity instantaneously entered human clinical trials (miR34a) and successfully meet the primary goals in the phase 1 study. This was the reason why we focused on the miRNA’s intrinsic abilities to tackle carcinogenesis, given the emerging number of evidence regarding miRNA taking control over certain key cancer genes [9]. Systemic delivery of miR34a by liposome-based approach in phase 1 clinical trials was later terminated due to the immune-related adverse effects in multiple cases that demand the improved alternative miR34a delivery system and by targeting advanced biological entity [8, 41]. miR34a mimics have shown tremendous anti-proliferative activity in a variety of different cancer models. Ample evidence has shown that the direct transfection of miR34a mimics failed to deliver encouraging results due to the non-specificity and instability of miR34a in serum. To overcome such limitations that have hampered the momentum of cancer therapeutics, researchers have recently spotlighted novel nano-delivery systems to vanquish such biopharmaceutical issues. These synthetic, multi-dynamic, and emerging nano-conjugation delivery systems have attracted a huge interest to support efficient transfection [42, 43]. Nanotechnology-based formulation of biopharmaceutical drugs is in the advanced phase of clinical trials by designing nanocarrier surfaces for different biological molecules including non-coding RNAs (miR34a). Also, a variety of nanocomposites has been investigated to deliver miR34a specifically to desired targeted locality or neighborhood of cell microenvironment, including silver, zinc, gold, PEG, PLGA, hyaluronic acid-chitosan nanoparticles, and silver bullets [44, 45] (Table 1). Indeed, lipid-based nanocarriers (cation liposomes) have revealed a key potential for nucleic acid delivery [46]. These cation liposomes have shown great ability to effectively interact with negative charge nucleic acid (noncoding RNAs) forming colloidal nanoparticles, called lipoplexes, that have been reported to be effectively taken up by the cell through endocytosis [47, 48]. A study was conducted in 2020 that involved the use of a polymer-based nanocomposite delivery system for the delivery of mir34a in patients with primary liver cancer. The study showed that the polymer-based delivery system was well-tolerated and resulted in the downregulation of several oncogenic pathways, including the c-Met and PDGF signaling pathways. In addition, the study showed evidence of antitumor activity, with some patients experiencing partial or stable disease response [49]. Furthermore, a phase I clinical trial conducted by miRagen Therapeutics involved the use of a nanoparticle-based delivery system for the delivery of mir34a in patients with hematological malignancies. The study showed that the nanoparticle-based delivery system was well-tolerated and resulted in the downregulation of several oncogenic pathways, including the BCL2 and STAT3 pathways. In addition, the study also depicted evidence of antitumor activity [50].

## Nanoscale delivery systems for miR34a in cancer therapy

MicroRNA (miRNA) based therapies have shown promising results in cancer treatment, and the use of nanocarriers for miRNA delivery can enhance their therapeutic efficacy. Researchers have used a combination of lipids, including phosphatidylcholine, cholesterol, and PEGylated lipids, to formulate the nanocomposites to evaluate *in vitro *and *in vivo* for their efficacy in reducing tumor growth in various preclinical studies of cancer treatment and inhibiting dysregulated cytokines. A few predominant types of nanocarriers that have been used for the delivery of miR34a in cancer treatment and their mechanisms of action are discussed in this review paper.

### Lipid-based nanoparticles (LNPs)

LNPs are spherical structures composed of lipids that can encapsulate miRNAs and deliver them to target cells. The mechanism of action of LNPs involves endocytosis, which facilitates the uptake of miRNAs into the cells. Lipid-based nanocomposites are designed to target cancer cells specifically. Once inside the cancer cell, the nanocomposite escapes the endosome to deliver the therapeutic cargo to the cytoplasm. This is achieved using pH-sensitive or membrane-disrupting lipids. Diffusion or enzymatic degradation of the lipid bilayer occurs within the cancerous cell to release therapeutic cargo (miR34a). The therapeutic cargo then interacts with its target molecules within the cancer cell, leading to the inhibition of tumor growth or induction of apoptosis. The lipid-based nanocomposite and any unbound therapeutic cargo are eventually eliminated from the body through various routes such as the kidneys and liver. LNPs have been shown to enhance miR34a expression and inhibit tumor growth in preclinical studies [51, 52]. In one of the latest studies published in the Journal of Controlled Release in 2022, researchers developed a lipid-based nanoparticle formulation for the delivery of a small molecule inhibitor of the protein kinase CK2 to treat pancreatic cancer [53]. It is observed that the lipid-based nanoparticles effectively delivered the CK2 inhibitor to the tumor site and significantly inhibited CK2 activity *in vitro* and *in vivo*. Furthermore, the nanoparticles were well-tolerated and did not show any signs of toxicity in the mice. The study demonstrates the potential of lipid-based nanoparticles as an effective and safe drug delivery system for the treatment of various cancer including pancreatic cancer [53].

### Polymeric nanoparticles (PNPs)

PNPs are polymeric-based nanoparticles that can encapsulate miRNAs and deliver them to target cells. PNPs can facilitate the uptake of miRNAs into the cells via endocytosis, and they can also protect miRNAs from degradation. PNPs have been shown to enhance the delivery of miR34a and inhibit tumor growth in preclinical studies [54]. Polymeric nanoparticles adopt same mode of action as describe earlier in lipid base nanocomposites, in addition it shows immunomodulation to modulate the immune response to cancer cells, leading to increased recognition and destruction of cancer cells by immune cells. Recently a group of researchers developed a polymeric nanoparticle-based drug delivery system for the treatment of pancreatic cancer. A polymer called poly(lactic-co-glycolic acid) (PLGA) is used to formulate the nanoparticles and loaded them with the chemotherapeutic drug gemcitabine. It was evaluated by *in vitro* and *in vivo* studies that PNPs efficacy in inhibiting tumor growth and reducing drug toxicity effectively by delivering gemcitabine to the tumor site significantly inhibited tumor growth in a mouse model of pancreatic cancer [55]. Furthermore, the PNPs were well-tolerated and did not show any signs of toxicity. The study demonstrates the potential of polymeric nanoparticles as an effective and safe drug delivery system for the treatment of pancreatic cancer and other solid tumors [56].

### Mesoporous silica nanoparticles (MSNs)

MSNs are porous silica-based nanoparticles that can encapsulate miRNAs and deliver them to target cells. MSNs can facilitate the uptake of miRNAs into the cells via endocytosis and can also provide sustained release of miRNAs. Mesoporous silica nanoparticles are designed to specifically target cancer cells via various mechanisms such as endocytosis. The anticancer agent (chemotherapy drug) is loaded into the pores of the MSN. The MSNs have a unique pore structure that allows for the controlled release of the drug over time, improving drug efficacy and reducing toxicity to healthy tissues. It released anticancer agents and induced immunogenic cell death in cancer cells, which can stimulate the immune system to recognize and destroy cancer cells. These nanoparticles also reported modulating the tumor microenvironment, promoting an anti-tumor immune response and inhibiting tumor angiogenesis, and eventually eliminating from the body through various excretory routes. MSNs have been shown to enhance miR34a expression and inhibit tumor growth in preclinical studies [57]. In a recent study, researchers utilize mesoporous silica nanoparticle-based drug delivery systems for the treatment of breast cancer. The researchers used MSNs loaded with the chemotherapy drug doxorubicin (DOX) and conjugated them with a tumor-targeting peptide called iRGD [58]. The MSNs were evaluated *in vitro* and *in vivo* for their efficacy in inhibiting tumor growth and reducing drug toxicity. It has been noted that the MSNs effectively delivered DOX to the tumor site and significantly inhibited tumor growth in a mouse model of breast cancer. In addition, the iRGD-conjugated MSNs showed enhanced tumor targeting and uptake, resulting in a higher accumulation of DOX in the tumor tissue [58, 59].

### Gold nanoparticles (GNPs)

GNPs are gold-based nanoparticles that can be functionalized with miRNAs and delivered to target cells. The mechanism of action of GNPs involves cellular uptake via endocytosis and the release of miRNAs into the cytoplasm. GNPs have been shown to enhance miR34a expression and inhibit tumor growth in preclinical studies [60]. Gold nanoparticles are also designed to specifically target cancer cells. After the successful delivery within the body of patient, they are taken up by cancer cells through various mechanisms such as receptor-mediated endocytosis. GNPs can be activated by external stimuli by using laser light or magnetic fields, leading to localized hyperthermia. Hyperthermia can induce cellular damage in cancer cells, while leaving surrounding healthy tissues relatively unharmed. GNPs can also generate ROS by the intervention of external stimuli, leading to oxidative stress in cancer cells. GNPs can be loaded with various drugs and targeted to cancer cells, improving drug delivery, and reducing toxicity to healthy tissues. GNPs have been shown to modulate the tumor microenvironment, promoting an anti-tumor immune response and inhibiting tumor angiogenesis [61]. In another recent study, the researchers investigated the mechanism of action of gold nanoparticles (GNPs) for the treatment of glioblastoma, a type of brain cancer. The researchers used GNPs conjugated with a tumor-targeting peptide and loaded with the chemotherapeutic drug temozolomide (TMZ). It is noted that the GNPs were able to penetrate the blood-brain barrier and accumulate in the tumor tissue, where they released the TMZ payload in a controlled manner. The GNPs also induced oxidative stress and DNA damage in the tumor cells, leading to cell death via apoptosis. It has been observed that the GNPs were able to modulate the immune response in the tumor microenvironment, leading to the recruitment of immune cells that further enhanced the anti-tumor effect of the treatment [62].

### Viral vectors

Viral vectors can be considered as nanocomposites because they consist of a nanoscale viral particle (typically between 20 and 100 nm in diameter) that has been engineered to deliver genetic material into cells. Lentiviruses and adenoviruses are viral vectors that can be used for miRNA delivery. The mechanism of action of viral vectors involves the integration of miRNAs into the host genome, leading to sustained expression of miRNAs. Viral vectors have been shown to enhance miR34a expression and inhibit tumor growth in pharmacological preclinical studies [63]. The viral vector is carefully chosen based on its ability to infect cancer cells efficiently and selectively. The vector is then modified to express miR34a and to reduce the risk of toxicity or immune response. The modified viral vector is then introduced into the body, typically through injection into the bloodstream or directly into the tumor. Infected cancer cells uptake the viral vector and miR34a is delivered into the cytoplasm. miR34a inhibits the expression of genes or targeted molecules, leading to apoptosis (cell death) of cancer cells. After successful action within the body, the viral vector is eliminated from the body through the excretory system. In a study published in 2020, researchers investigated the use of a viral vector to deliver miR34a to cancer cells in patients with malignant pleural mesothelioma, a type of cancer that affects the lining of the lungs. The researchers used a type of viral vector called a lentivirus to deliver a modified form of miR34a to the tumor cells. The miR34a was designed to suppress the expression of a protein called c-Met, which is involved in the growth and spread of cancer cells. It is observed that the lentivirus-delivered miR34a was able to significantly reduce the growth of mesothelioma tumors in mouse models. The therapy was also found to be safe and well-tolerated in phase I clinical trial, with some patients experiencing disease stabilization or regression [64]. The published research related to preclinical and clinical studies demonstrates the potential of various nanocomposites as an effective drug delivery system for the treatment of breast cancer and other solid tumors. Furthermore, the use of tumor-targeting peptides may enhance the therapeutic efficacy of the nanoparticles by improving their tumor specificity. Furthermore, the ability of GNPs to modulate the immune response in the tumor microenvironment may enhance the therapeutic efficacy of the nanoparticles by promoting an anti-tumor immune response. The use of miR34a-delivering lentiviral vectors could potentially be extended to other types of cancer, particularly those that overexpress c-Met or other targetable genes. This review highlights the potential of nanocomposite-mediated delivery of miRNAs as a promising approach for cancer treatment, particularly for cancers that are difficult to treat with traditional therapies.

**Table 1:** Clinical applicability of miR34a bioconjugates in cancer

MiR-34a Bioconjugate	Targeted Cancer / Type of study	Results	Ref
miR34a + gold nanoshells	Breast cancer Triple-negative breast cancer (TNBC) cells *In vitro*	↓cancer cell viability ↓proliferation, ↓migration	[65]
MUC1-AgNC_m_-miR-34a	Breast cancer MCF-7 cells *In vitro*	↓ Bcl-2, ↓Cyclin D1, ↓CDK6	[66]
PEGylated-thymoquinone-nanoparticle-miR-34a	Breast cancer Human mammary carcinoma cell lines MCF-7, HBL-100 *In vitro*	↓cytoskeletal actin polymerization, ↓migration	[67]
cRGD-PEG/miR-34a liposomes	Breast cancer MDA-MB-231 cells *In vitro*	↓CD44^+^/CD24^−/low^ cancer stem cells ↑inhibition of tumor cells, ↓cell proliferation, ↓migration, ↓invasion	[68]
SNALPs- miR-34a- transferrin	Multiple myeloma *In vitro* MM cell lines *In vivo* mouse model with SKMM-1 tumor xenografts	↓ tumor cell growth ↑survival of mice	[69]
DOX-miR-34a co-loaded HA-CS NPs	Breast cancer *In vitro* Triple negative and mesenchymal-type breast cancer cell lines *In vivo* mice	↓Bcl-2, ↓migration ↓invasion of breast cancer cells by targeting NOTCH-1 signaling	[44]
miRNA-34a/PLI/HA	Triple negative breast cancer cell lines *In vitro* TNBC cells	↓Bcl-2, ↓cancer cells viability	[70]
miR-34a - GD_2_ antibody-silica NPs	Pancreatic cancer MIA PaCa-2 cells Panc-1 cell lines *In vitro*	↓ tumor cell growth, ↓vascularization ↑TIMP2, ↑apoptosis	[71]
EGCG-capped AuNP- miR-34a and let-7a	Hepatocarcinoma cells * In vitro*	↓ C-Myc, ↑caspase-3	[68]
miR-34a - poly(lactic-co-glycolic acid) NPs	Breast cancer *In vitro* TNBC cells	↓ cell proliferation, ↓migration, ↓invasion, ↓NOTCH	[69]
APA-miRNA–siRNA polyplexe	Pancreatic cancer *In vitro* MiaPaCa2 cells	antitumor effect, ↑ C-Myc	[72]
cationic albumin (nanocarrier)- miRNA-34a- docetaxel (DTX)	Breast cancer *In vivo* 4T1-tumor bearing mouse model	↓Bcl-2, ↓cancer cell viability, ↑apoptosis, ↑cytotoxicity	[70]
miR34a-volasertib (B16727)-PEG-B-PAEBEA	Pancreatic cancer *in vivo* BALB/c nude mice with MIA PaCa-2 xenograft models	↓ tumor volume, ↑antiproliferative activity, ↓C-Myc	[71]
PPP/miR-34a	Gastric cancer *In vitro* Human gastric cancer cell lines *In vivo* mice	↓proliferation, ↓cell migration, ↓invasion, ↓ NOTCH	[73]

Abbreviations and symbols: ↑increase, ↓decrease, mucin 1 (MUC1), fluorescent silver nanocluster (AgNC), MicroRNA-34a (miR34a), Cyclic RGD (cRGD)- Polyethylene glycol-modified (PEGylated), Stable nucleic acid lipid vesicles (SNALPs), doxorubicin (DOX) hyaluronic acid (HA)-chitosan (CS), nanoparticles (NPs), chitosan-hyaluronic acid nanoparticles (GCHN), gadoliniu (Gd), Poly-L-Lysine graft imidazole (PLI), Epigallocatechin gallate (EGCG), Gold nanoparticles (AuNPs), poly(ethylene glycol)–poly[aspartamidoethyl(p-boronobenzyl)diethylammonium bromide] (PEG-B-PAEBEA), MM (multiple myeloma)

Many reports highlighted the effectiveness of stable nucleic acid lipid particles (SNALPs) in several *in vivo* multiple myelomas xenografts pharmacological studies [74]. The worth noting is that SNALPs as a delivery system hold great promise for significant tumor growth inhibition. SNALP miR-34a induced anti-multiple myeloma therapy reveals that there is a likeness increase in intratumor miR-34a proportion and a gradual decrease in NOTCH1 expression. Recently, it has been observed that SNALP encapsulating miR34a to treat myeloma cancer significantly boosts the delivery of miR34 and increases survival percentage in mice, whose SNALPs are nano-conjugated with Transferrin “Tf” [69].

Neutral lipid emulsion (NLE) is used for safe and effective therapeutic delivery of synthetic microRNA (miRNA) mimics to non-small cell lung cancer (NSCLC). NLE-encapsulated miR-34a introduced systemically by intravenous injections in mice shows an overall reduction in tumor growth. Studies have revealed that these particles form fewer aggregates than conventional cationic lipids in biofluids, and do not adhere to the endothelium taken up by macrophages [75]. In this field, the most encouraging procedure in miRNA therapeutics is the systematic delivery of miRNA mimics or inhibitors. However, the main problem in miR34a replacement therapy in lung cancer treatment is its bioavailability in liver cells. Thus, for effective delivery of MicroRNA-34a (miR-34a), a potent tumor suppressor in NSCLC was encapsulated into S6 aptamer-conjugated dendrimer to form targeted gene delivery nanoparticles (PAM-Ap/pMiR-34a NPs) for the treatment of lung cancer. Poly-amidoamine dendrimers are “smart” carriers with the potential to serve as intracellular gene delivery vehicles conjugated to S6 aptamer. It was reported that PAM-Ap/pMiR-34a NPs have a significantly higher cell uptake and transfection efficiency than the previous formulation. Also, this bioconjugate nanoparticle could significantly increase the percentage of early and late apoptotic cells and increase the regulation of tumor suppressor oncogenes BCL-2 and p53 *in vitro* [76].

Metal oxide nanoparticles are well known for their ability to induce a cytotoxic response in cancer treatment either alone or in combination with photocatalytic therapeutic strategies or some anticancer drugs. Among them, ZnO nanoparticles are used in advanced biomedical and therapeutic applications, including targeted delivery of anti-cancer drugs. Specifically, the zinc oxide NPs activity has been reported against HepG2 (liver cancer) and MCF-7 (breast cancer) cells in a dose-dependent manner, being able to lower the levels of apoptotic markers p53, Bax, Bcl-2, and caspase-3. Another study using zinc oxide NPs in conjugation with asparaginase reported a decrease in MCF7 cell viability. More recently, the biotinylated porous hexagonal nanodisc of zinc oxide (PZHD) was reported for the first time for targeted delivery of the chemotherapeutic drug doxorubicin on MCF7 breast cancer cells with such antitumor effects greatly holding a future therapeutic intervention for the treatment of cancer [77].

Regarding the prostate cancer, many advanced drugs have been approved to date for the treatment of metastatic prostate cancer (PCa), but life expectancy is still very low. For example, in a study on xenograft mouse model for PCa bone metastasis, chitosan NPs coated miR34a led to the downregulation of multiple gene products (MET, AXL, and C-Myc) that play a significant role in PCa progression and metastasis, not only inhibiting PCa growth but also preserving bone integrity by MiR-34a-induced non-canonical autophagy and apoptosis, lastly leading to tumor growth inhibition [78]. Recent studies have also stressed that the miR34a downregulation has been associated with chemo-resistant behavior against various effective drugs, including paclitaxel. For example, it was observed that PEG-PCD micelles-based co-delivery of paclitaxel and rubone (2’-hydroxy-2,4,4’,5,6’-pentamethoxychalcone) upregulated miR34a and acted as a potential killer of both tumor cell and cancer stem cells growth. In nude mice, the systemic administration of these micelles inhibited PCa proliferation and reversed the expression of cyclin D1, E-Cadherin, SIRT1, and miR34a. Thus, rubone is currently viewed as an effective modulator of miR34a that enhanced the therapeutic application of chemo-resistant PCa drugs [79].

Overall, these studies demonstrate the potential of nanocomposite delivery systems for the efficient delivery of mir34a in clinical settings. However, further research is needed to optimize these systems for specific diseases and to improve their efficacy and safety profiles.

## Conclusions and future perspectives

miRNA-34a controls the expression of a plethora of target proteins involved in tumorigenesis. The miR34a replacement therapeutic approach along with advanced selective nanotechnology-based delivery systems act as a magnificent platform holding promise for the onco-suppression of miRNA, ultimately avoiding tumor heterogeneity. miR34a plays a decisive role in apoptosis, cell differentiation, and cell cycle. However, miR34a deficiency alone is not strong enough to induce cancer onset in most cases, while the contributing effect of p53-mediated miR34a triggers oncogenesis in various cancers. With the advances in the field of nanotechnology, researchers were capable to deepen knowledge and boost targeted delivery strategy and miR34a-mediated suppression activity, so that the antiproliferative potential of miR34a derives us to further enhance its permeability and stability in the biological environment by coupling miR34a with nano delivery systems [80, 81].

Various preclinical studies *in vivo*, in mice models have revealed that nano-conjugate delivery systems could be the potential game-changer, given their excellent ability to improve the bioavailability of potential drugs and synthetic biomolecules, including miR34a [58–60]. Besides these advances, the technological improvements have also offered advanced material designing for drug development with specialized surfaces and smart sensing ability capable to enhance the anticancer biological mechanisms, including apoptosis and inflammation. Thus, both new biomarkers and materials with promising potential can be designed for drug delivery, and will shape the future of the modern healthcare industry for a wide range of diagnosis. Moreover, in the future, miR34a-based targeted therapy using nanocomposite delivery systems could become a powerful tool for cancer treatment in terms of personalized medicine, combination therapy, and non-invasive treatment. In personalized medicine, nanocomposite delivery systems can be tailored to deliver miR34a specifically to cancer cells, while sparing healthy cells. In combination therapy, miR34a-based targeted therapy could be combined with other therapies, such as chemotherapy or immunotherapy, to enhance their efficacy. And in non-invasive treatment, nanocomposite delivery systems can be delivered non-invasively, such as through inhalation or injection, reducing the need for invasive surgical procedures.

The future of nanocomposites in cancer treatment looks promising as they offer several advantages such as targeted drug delivery, improved pharmacokinetics, reduced toxicity, and the ability to overcome drug resistance. With continued research and development, nanocomposites have the potential to revolutionize cancer treatment by providing more effective and personalized therapies. However, there are still challenges to overcome such as optimizing the design and manufacturing of nanocomposites, ensuring their safety and efficacy, and addressing regulatory and ethical concerns. Nonetheless, the field of nanocomposites holds great promise for the future of cancer treatment.

## Data Availability

Not Applicable.
